# Comparative Evaluation of the Effects of Oral Anti-hyperglycemic Agents and an Ayurvedic Hepatoprotective Agent in a Rat Model of Non-alcoholic Fatty Liver Disease (NAFLD)

**DOI:** 10.7759/cureus.99891

**Published:** 2025-12-22

**Authors:** S. Shamsher S Kalra, Balakrishnan Sadasivam, Ahmad Najmi, Jai K Chaurasia

**Affiliations:** 1 Pharmacology, All India Institute of Medical Sciences, Bhopal, IND; 2 Pathology and Laboratory Medicine, All India Institute of Medical Sciences, Bhopal, IND

**Keywords:** dapagliflozin, enzyme-linked immunosorbent assay (elisa), glimepiride, histopathology, liv52, metabolic dysfunction-associated steatotic liver disease (masld), nafld activity score, pioglitazone, rat model, vildagliptin

## Abstract

Introduction

Metabolic dysfunction-associated steatotic liver disease (MASLD) is a chronic liver disease characterized by the accumulation of triglycerides (TG) in the liver, and histopathological findings include simple fatty liver, steato-hepatitis, fibrosis, and ultimately cirrhosis. Our study assessed the efficacy of oral anti-hyperglycemic agents (glimepiride, vildagliptin, and dapagliflozin) and an ayurvedic medicine (Liv52) in an induced MASLD rat model both biochemically and histopathologically.

Materials and methods

It was a pre-clinical experimental study whereby Sprague-Dawley rats (n = 36, male = female, 18-20 weeks old, weight 150-180 g) acclimatized in an animal house facility (12-hour light/dark cycle) were used. Baseline total cholesterol (TC), TG, aspartate aminotransferase (AST), and alanine aminotransferase (ALT) enzyme-linked immunosorbent assay (ELISA) was done, and then, all animals were divided into six groups (M = F) of six rats each (negative control - only high-fat diet, glimepiride group, vildagliptin group, dapagliflozin group, Liv52 group, and positive control - pioglitazone group, respectively) and fed with high-fat diet and water ad libitum for six weeks. Repeat TC, TG, AST, and ALT estimations were done to confirm model induction. Thereafter, each group was given its pertinent drug for four weeks by oral gavage along with a high-fat diet + water ad libitum. Final TC, TG, AST, and ALT estimations were done followed by the sacrifice of all rats, liver isolation, and histopathological examination for the NAFLD activity score (NAS).

Results

High-fat diet administration led to deranged TC, AST, and ALT values in the Sprague-Dawley rats, thereby confirming model induction. The glimepiride group exhibited reduced AST, ALT, and NAS along with increased TC and TG levels. The vildagliptin group exhibited reduced TC, AST, ALT, and NAS along with increased TG levels. The dapagliflozin group exhibited reduced TC, TG, and AST along with increased ALT levels and no change in NAS. The Liv52 group exhibited reduced TC and NAS along with increased TG and AST levels and no changes in ALT levels. Also, four rats of the Liv52 group died within a week of dosing, and their liver histopathology showed focal necrosis, inflammation, and congestion. Finally, the pioglitazone group (positive control) exhibited reduced TC, AST, and NAS along with increased TG and ALT levels.

Conclusion

We conclude that glimepiride, which exhibited AST and ALT reductions along with a four-point NAS reduction, offers itself as a promising drug candidate for human clinical trials for MASLD. Vildagliptin, which exhibited TC, AST, and ALT reductions but a less promising one-point NAS reduction, can also be tried in human clinical trials for MASLD.

## Introduction

Non-alcoholic fatty liver disease (NAFLD) is an emerging global health concern whose prevalence is nearly 30% of the global adult population. This is corroborated by an increased prevalence of obesity worldwide [1]. Histopathologically, NAFLD is characterized by hepatic fat accumulation, and its spectrum ranges from simple fatty liver, non-alcoholic steato-hepatitis (NASH), fibrosis, and ultimately cirrhosis. Liver cirrhosis has an increased propensity to progress toward hepatocellular carcinoma [2].

Delving into the pathogenesis of NAFLD, insulin resistance and metabolic syndrome are closely intertwined with it. Hence, NAFLD is also believed to be a hepatic manifestation of metabolic syndrome. Therefore, the newer nomenclature of NAFLD is MASLD, i.e., metabolic dysfunction-associated steatotic liver disease [3].

Despite intensive research over the past years, NAFLD treatment still remains a therapeutic challenge. The two-hit theory suggests that hepatic fat accumulation sensitizes its hepatocytes, leading to hepatocellular inflammation, damage, and ultimately fibrosis. The second hits usually are microsomal cytochrome P450 induction, peroxidation of lipids, mitochondrial abnormalities, imbalance between adipokines and cytokines (namely, tumor necrosis factor α (TNF-α), interleukin-6, adiponectins, leptin, and resistin), hepatic cholesterol accumulation, gut lipopolysaccharides, free fatty acid toxicity, or activation of innate immunity. The net effect of these two hits is hepatocytic cell death. Longstanding liver injury coupled with regenerative responses leads to stellate cell activation and fibrotic changes [4].

Characterizing NAFLD histopathologically is done by liver steatosis > 5% (macrovesicular/microvesicular/mixed). Adults commonly present with macrovesicular steatosis either in Zone 3 or in a pan-acinar fashion [2].

NASH is histopathologically characterized by hepatocellular ballooning (usually Zone 3) and lobular/portal inflammation (involving macrophages, eosinophils, monocytes, and lymphocytes). It is also accompanied by apoptosis of hepatocytes and is a good predictor of NASH severity [2]. Hence, current diagnostic paradigms place histopathology as the gold standard for accurate assessment of degrees of hepatic steatosis, inflammation, and fibrosis.

The pathophysiology and treatment of NAFLD can be understood by replicating the disease in suitable animal models. An ideal animal model faithfully portrays all aspects of the disease etio-pathogenesis and the histopathological findings as seen in human beings. Substantial efforts have been made in this regard so as to design an animal model that incorporates at least the fundamental pathophysiologic and histological features of NAFLD. The basic tenets of an animal model are reproducibility, reliability, simplicity, affordability, and technical availability coupled with minimum disadvantages. To date, rats and mice have been the go-to animals for modelling NAFLD. The variety of these models ranges from genetic models and high-fat diet (HFD) models to choline-methionine diet-deficient models. These models exhibit hepatic steatosis as a common feature, along with progression to steato-hepatitis and fibrosis in some models [5].

Animal studies per se are conducted on well-defined groups with regard to species, strain, gender, weight, age, lab conditions, diet, and other factors. This experimental homogeneity is contrasted to the heterogeneity seen in human beings even if they are matched with respect to age, gender, place, weight, and ethnicity. This is due to the high variability of genetic background, physical activity, concomitant medications, dietary practices, co-morbidities, individual lifestyle, and other relevant confounding factors. So, even with the best animal modelling of the disease, the extrapolability of their results to human ailments is limited. Additionally, rodents have peculiar characteristics distinct from humans that can alter the NAFLD etio-pathogenesis, thereby further hindering the interpretability of the accumulated results. Hence, NAFLD animal modelling and drug experimentation should be verified with suitable human clinical trials for achieving a complete understanding and extrapolability of the results.

Moving further into this arena, the current treatment options for NAFLD in the modern medicine/allopathic domain are weight loss regimens (Mediterranean/DASH (Dietary Approaches to Stop Hypertension) diet) for achieving 3%-5% weight loss whereby 10% weight loss is considered optimal; physical activity of ~30 minutes of aerobic exercise for at least five days per week; smoking cessation; abstinence from alcohol consumption; hepatitis A and B vaccinations; glucagon-like peptide-1 (GLP-1) agonists: semaglutide and liraglutide; dual glucose-dependent insulinotropic polypeptide (GIP) and GLP-1 receptor agonist: tirzepatide; thyroid hormone receptor-beta (THR-β) agonist: resmetirom; bariatric surgery; and thiazolidinedione: pioglitazone. Out of the aforementioned pharmacotherapies, only resmetirom, semaglutide, and pioglitazone have an oral route of drug administration [6].

In the domain of traditional/alternative medicine (AYUSH (ayurveda, yoga and naturopathy, Unani, Siddha, and homeopathy)), the treatment of NAFLD has been tried with a multitude of approaches, but the most popular commercially available formulation is Liv52. It is a combination of herbal products like himsra (*Capparis spinosa*), kasani (*Cichorium intybus*), mandur bhasma, kakamachi (*Solanum nigrum*), arjuna (*Terminalia arjuna*), kasamarda (*Cassia occidentalis*), biranjasipha (*Achillea millefolium*), jhavuka (*Tamarix gallica*), bhringraj (*Eclipta alba*), bhumyamalaki (*Phyllanthus amarus*), punarnava (Boerhavia diffusa), guduchi (*Tinospora cordifolia*), daruharidra (*Berberis aristata*), mulaka (*Raphanus sativus*), amalaki (*Emblica officinalis*), chitraka (*Plumbago zeylanica*), vidanga (*Embelia ribes*), haritaki (*Terminalia chebula*), and parpata (*Fumaria officinalis*). This ayurvedic proprietary medicine is claimed to protect the liver against hepatotoxins, guard it against jaundice and hepatitis A and B, stimulate overall body growth and appetite, and also improve the digestive process [7].

In addition to the aforementioned treatment avenues for NAFLD encompassing the entire gambit of modern and traditional medical domains, research has been ongoing for better pharmacotherapies with convenient routes of administration to aid in patient compliance and well-being. These include the following.

Glimepiride

In an open-label, randomized trial, it significantly reduced the NAFLD activity score (NAS) after 48 weeks in participants with liver biopsy specimen-confirmed NAFLD and type 2 diabetes. The percentage of participants with improvement in the fibrosis stage was 35%, similar to that of participants with obeticholic acid treatment and greater than that of placebo participants [8].

Vildagliptin

Endoplasmic reticulum (ER) stress was alleviated to a certain extent by treatment with vildagliptin in HFD mouse models of NAFLD. Treatment group mice showed lower increases in liver weight/body weight and liver triglycerides (TG) than control mice. The HFD-induced expression of ER stress indicators, such as binding immunoglobulin protein (BiP), protein kinase R-like ER kinase (p-PERK), translation initiation factor-2α (p-eIF2), inositol-requiring enzyme type-1 (p-IRE1), and X-box binding protein-1 (xBP-1), was also inhibited by vildagliptin [9].

Dapagliflozin

It lowers blood alanine aminotransferase (ALT) level, liver weight, TG content, and NAS, indicating that it can ameliorate NASH in db/db mice. The elevated expression of sterol regulatory element binding protein-1c (SREBP-1c) was also significantly reduced [10].

The primary objective of our study was to assess the efficacy of oral anti-hyperglycemic agents (glimepiride, vildagliptin, and dapagliflozin) and an ayurvedic medicine (Liv52) in the treatment of NAFLD in a rat model as compared to a positive control (PC) and a negative control (NC) both biochemically and histopathologically. Secondary objectives were to quantify the extent of liver damage biochemically in a NAFLD Sprague-Dawley rat model and to quantify the grade of hepatic histopathological improvement in the intervention arms as compared to the PC and NC.

## Materials and methods

Study design

Our study was a pre-clinical experimental study.

Animals and randomization

Male and female Sprague-Dawley rats were used for the study. The animals were housed in polypropylene cages of 40 × 20 × 15 cm; up to six animals were kept in one cage. They were kept in standard laboratory conditions under a 12-hour light and dark cycle. Inclusion criteria were age of 18-20 weeks and weight of 150-200 g, whereas exclusion criteria were rats with any known hepatic disorder. Eighteen male and 18 female rats were randomly selected from the available stock of the Central Animal House facility, which were then equally divided into the six groups of six rats each (three male and three female).

Experimental model of MASLD

HFD was used to induce MASLD in the rats. The HFD contained about 350-450 kcal/100 g with 40%-60% as fat and the remaining as an appropriate distribution of carbohydrate and protein proportions. The fat content of the diet was provided by hydrogenated vegetable cooking oil (Dalda). Animals showing deranged liver function test parameters after six weeks of HFD administration were considered as having developed MASLD.

Drugs/materials required

The drugs/materials required include the following: HFD (formulated with 40%-60% fat, total weight: 65 kg adequate for feeding 36 rats at a rate of 20 g/day for 70 days of total experimental period); tablets pioglitazone 15 mg (USV, India), glimepiride 1 mg (Sanofi, India), vildagliptin 50 mg (Novartis, India), dapagliflozin 5 mg (Medley Pharmaceuticals, India), and Liv52: double strength (Himalaya Wellness, India); reagents/kits: validated commercial enzyme-linked immunosorbent assay (ELISA) kits for the assessment of biochemical parameters selected for this study (aspartate aminotransferase (AST), ALT, total cholesterol (TC), and TG), procured from Krishgen Biosystems (India); and a nano-spectrophotometer with an ELISA plate reader (available in the Department of Pharmacology).

Experimental procedure

Phase 1

Phase 1 includes the measurement of baseline AST, ALT, TC, and TG in the rats and induction of MASLD by the administration of HFD for six weeks. Repeat estimation (AST, ALT, TC, and TG) was performed at the end of six weeks to confirm MASLD induction in the rats. The animals were monitored every day, and any fatality was documented.

Phase 2

Phase 2 includes the administration of the drugs by oral gavage in the six groups of six rats each along with HFD for four weeks (Liv52: 70 mg/kg/day, glimepiride: 0.18 mg/kg/day, pioglitazone (PC): 2.6 mg/kg/day, vildagliptin: 8.8 mg/kg/day, dapagliflozin 0.9 mg/kg/day, and normal saline (NC)). The animals were monitored every day, and any fatality was documented. This was followed by the final estimation of AST, ALT, TC, and TG in all groups followed by the sacrifice of all the rats (following the humane carbon dioxide inhalation euthanasia approach), liver isolation, formalin fixation, hematoxylin-eosin staining, and subsequent histopathological analysis.

Histopathological grading/scoring of MASLD

NAS is defined as the unweighted sum of the scores for steatosis (0-3), lobular inflammation (0-3), and ballooning (0-2), thus ranging from 0 to 8. Fibrosis is not included in this semi-quantitative grade, since fibrosis is generally less reversible and thought to be the result of disease activity rather than a potential driver lesion. The NAS was recently validated and shown to be reproducible and easy to use and has been used in numerous clinical trials and cross-sectional studies [11].

Sample size estimation

Sprague-Dawley rats were required for the two phases of the study. Using the resource equation method for calculating the sample size in animal studies, six animals per group were required, including 10%-15% in-study mortality. Hence, a total of 36 animals were required in conducting phases I and II of the study [12].

Statistical analysis

Data were displayed using appropriate tabular representations, and results were calculated using frequencies, proportions, and mean with standard deviation. Normality testing was done by the Shapiro-Wilk test. A paired t-test was used for intra-group analysis of parametric data, and the Wilcoxon signed-rank test was used for non-parametric data. A “p”-value of less than 0.05 was considered significant. Data were entered and stored in Microsoft Excel (Microsoft Corp., Redmond, WA, US). GraphPad version 10.6.1 (Dotmatics, Boston, MA, US) was used for statistical analysis.

Ethical considerations

This study was undertaken following the provision of permission from the Institutional Animal Ethics Committee (IAEC), All India Institute of Medical Sciences, Bhopal (vide letter of permission number: AIIMS/BPL/IAEC/2024/056 dated February 12, 2024). The study was conducted in accordance with the CCSEA (Committee for Control and Supervision of Experiments on Animals) guidelines with regard to animal handling, care, and management.

Blinding

The study followed a double-blind pattern whereby the lab technician doing the biochemical analyses and the histopathologist observing the liver slides for NAS were blinded about the group of rats they were analyzing.

Study site

The study was conducted at the Department of Pharmacology, All India Institute of Medical Sciences, Bhopal. The animal experimentation was done in the Central Animal House facility, which is equipped with all standard requisites for animal housing, feeding, and handling. Biochemical analysis was done in the Department of Pharmacology. Histopathological analysis was done by the Department of Pathology, All India Institute of Medical Sciences, Bhopal.

## Results

First of all, we did a baseline ELISA of the liver function test (AST and ALT) as well as lipid profile (TC and TG) parameters in all 36 rats to confirm normal physiology before the start of the experiment. However, only 28 rats survived till the end of the experiment; mentioned below are the baseline biochemical parameters of those 28 rats (Table 1). ELISA kits’ limit of detection is as follows: TC: 0.262 mg/dL, TG: 1.045 mg/dL, AST: 0.2 U/L, and ALT: 0.02 U/L, respectively.

**Table 1:** Baseline biochemical parameters (n = 28) NC: negative control (normal saline); L: Liv52; G: glimepiride; V: vildagliptin; D: dapagliflozin; PC: positive control (pioglitazone); SD: standard deviation; TC: total cholesterol; TG: triglyceride; AST: aspartate aminotransferase; ALT: alanine aminotransferase

Rat	TC (mg/dL)	TG (mg/dL)	AST (U/L)	ALT (U/L)
NC 1	140	129	41	0
NC 2	185	121	8	0
NC 3	133	154	0	0
NC 4	48	127	0	0
NC 5	231	119	0	0
NC 6	165	119	0	0
L 5	40	125	11	0
G 2	171	138	0	0
G 3	137	122	0	0
G 4	147	128	8	0
G 5	53	139	29	0
G 6	43	123	0	0
V 1	111	129	0	0
V 2	258	131	0	0
V 3	138	150	0	0
V 4	181	122	0	0
V 5	201	129	24	3
V 6	74	128	18	6
D 1	180	101	0	0
D 3	0	124	0	0
D 4	140	129	0	0
D 5	144	120	0	0
D 6	127	123	1	0
PC 1	138	129	0	0
PC 2	127	139	0	0
PC 3	95	128	0	6
PC 4	0	127	0	0
PC 6	0	140	0	0
Mean ± SD	121.67 ± 68.1	128.32 ± 10.2	5 ± 10.5	0.53 ± 1.6

The normal value of these parameters from other studies is given in Table 2 [13,14].

**Table 2:** Reference values of biochemical parameters TC: total cholesterol; TG: triglyceride; AST: aspartate aminotransferase; ALT: alanine aminotransferase

Rat	TC (mg/dL)	TG (mg/dL)	AST (U/L)	ALT (U/L)
Sprague-Dawley	113.99 ± 2.2	76.13 ± 2.4	68.61 ± 20.7	32.72 ± 19.5

These findings suggest that the baseline TC, TG, AST, and ALT values obtained in our study were well within comparison to the baseline values obtained by other researchers across the globe. Interestingly, there is a wide heterogeneity in these reference values as reported by different studies.

After baseline ELISA estimations, we proceeded with the HFD administration for the stipulated six-week duration. On the completion of the six-week induction phase, we again carried out ELISA estimations on all rats; two rats (one of the Liv52 group and one of the PC: pioglitazone group) had died in these six-week duration while only 28 rats survived till the end of the study. Therefore, we hereby present the induction sampling values of TC, TG, AST, and ALT for the 28 rats that made it till the end of our study (Table 3).

**Table 3:** Post-HFD administration biochemical parameters (n = 28) after six weeks NC: negative control; L: Liv52; G: glimepiride; V: vildagliptin; D: dapagliflozin; PC: positive control (pioglitazone); SD: standard deviation; TC: total cholesterol; TG: triglyceride; AST: aspartate aminotransferase; ALT: alanine aminotransferase; HFD: high-fat diet

Rat	TC (mg/dL)	TG (mg/dL)	AST (U/L)	ALT (U/L)
NC 1	97	124	0	0
NC 2	288	128	0	0
NC 3	154	146	25	0
NC 4	330	140	9	16
NC 5	120	135	52	5
NC 6	207	115	52	8
L 5	206	121	42	0
G 2	0	121	43	0
G 3	0	115	52	0
G 4	77	115	52	0
G 5	64	124	52	5
G 6	274	118	52	0
V 1	212	122	14	8
V 2	57	128	0	0
V 3	211	122	11	0
V 4	84	117	9	0
V 5	236	117	20	1
V 6	183	114	3	12
D 1	226	147	0	0
D 3	184	140	0	1
D 4	218	124	53	7
D 5	205	138	53	0
D 6	199	129	21	0
PC 1	286	122	31	0
PC 2	164	123	48	0
PC 3	281	116	41	2
PC 4	4	120	44	1
PC 6	353	118	40	0
Mean ± SD	175.71 ± 98.2	124.96 ± 9.6	29.25 ± 21.02	2.35 ± 4.2

Thereafter, we compared the induction sampling biochemical parameters’ values to those of the baseline after normality testing with the Shapiro-Wilk test followed by a paired t-test for parametric data and the Wilcoxon signed-rank test for non-parametric data. The results of the analysis are as follows (Table 4).

**Table 4:** Comparison of baseline and post-HFD biochemical parameters p < 0.05 in bold. The means of TC, TG, AST, and ALT values of all 28 rats as a whole were compared between baseline and post-HFD administration for six weeks (as during the first six weeks, no drug intervention was given in any group; the groups were only segregated into six groups of six rats each). Paired t-test was applied for TC values' comparison (as of normal data distribution), and the Wilcoxon signed-rank test was applied for TG, AST, and ALT values' comparison (as of non-normal distribution). TC: total cholesterol; TG: triglyceride; AST: aspartate aminotransferase; ALT: alanine aminotransferase; HFD: high-fat diet; SD: standard deviation

Biochemical parameters	Baseline values (mean ± SD)	Post-HFD values (mean ± SD)	Shapiro-Wilk normality test p-value	Paired t-test p-value	Wilcoxon signed-rank test p-value
TC (mg/dL)	121.67 ± 68.1	175.71 ± 98.2	0.91	0.04	-
TG (mg/dL)	128.32 ± 10.2	124.96 ± 9.6	0.005	-	0.1
AST (U/L)	5 ± 10.5	29.25 ± 21.02	0.02	-	0.0002
ALT (U/L)	0.53 ± 1.6	2.35 ± 4.2	0.000006	-	0.02

Comparison of the post-HFD with baseline biochemical parameters using the paired t-test/Wilcoxon signed-rank test as per the normality of the data revealed that “HFD administration led to deranged TC, AST, and ALT values in the Sprague-Dawley rats,” thereby confirming model induction. After successful MASLD model induction, the rats were given the individual drugs by oral gavage along with continued HFD for a further duration of four weeks.

Just within a week of drug dosing, four rats of the Liv52 group died, while one rat each of the glimepiride and dapagliflozin groups died in the fourth week of drug dosing. Histopathological examination of liver samples of the deceased rats of the Liv52 group (n = 4) showed the following findings (Table 5).

**Table 5:** Histopathological examination of the liver of deceased rats (Liv52 group) L: Liv52 The fifth rat (L4) died during the induction phase, that is, during the first six weeks whereby only HFD was administered; hence, its death was not attributable to Liv52, which was given to the remaining five rats after model induction. Hence, the histopathology of the fifth rat was not done.

Rat	Histopathological findings
L 1	Congested central vein, dilated and congested sinusoids, mild steatosis, and no hepatocyte ballooning
L 2	Microvesicular steatosis, normal sinusoids, congested central vein, peri-portal inflammation, no necrosis, and hepatocyte ballooning
L 3	Dilated sinusoids, congested central veins, grade 2 steatosis, mild peri-portal inflammation, mild necrosis, and no hepatocyte ballooning
L 6	Focal centri-lobular necrosis, congested central vein, dilated sinusoids, mild inflammation, microvesicular steatosis, and no hepatocyte ballooning

Final blood sampling and ELISA were done on 28 rats at the end of four weeks to ascertain the effects of individual drugs on the Sprague-Dawley rat MASLD model. The results of that biochemical analysis are as follows (Table 6).

**Table 6:** Post-intervention biochemical parameters (n = 28) after 10 weeks (six-week induction phase and four-week intervention phase) NC: negative control; L: Liv52; G: glimepiride; V: vildagliptin; D: dapagliflozin; PC: positive control (pioglitazone); TC: total cholesterol; TG: triglyceride; AST: aspartate aminotransferase; ALT: alanine aminotransferase

Rat	TC (mg/dL)	TG (mg/dL)	AST (U/L)	ALT (U/L)
NC 1	78	129	14	0
NC 2	93	138	52	6
NC 3	109	146	0	9
NC 4	105	157	0	3
NC 5	248	148	0	9
NC 6	292	117	0	0
L 5	25	128	44	0
G 2	206	113	0	0
G 3	270	125	0.5	0
G 4	132	132	27	0
G 5	243	145	5	0
G 6	2.5	154	21	2
V 1	0	143	7	0
V 2	0	118	0	0
V 3	0	118	0	0
V 4	404	104	0	0
V 5	231	134	0	0
V 6	288	151	0	0
D 1	74	119	25	0
D 3	81	130	0	0
D 4	117	123	0	7
D 5	240	119	0	1
D 6	3	135	0	4
PC 1	0	112	53	2
PC 2	195	135	12	5
PC 3	271	113	22	0
PC 4	101	141	22	0
PC 6	0	158	0	0

Thereafter, comparisons were made in individual groups for changes in biochemical parameters (if any) brought out by the drug intervention. The Wilcoxon signed-rank test was performed to study intra-group pre/post drug dosing variations. The results of that analysis are presented in Tables 7, 8.

**Table 7:** Intra-group pre/post dosing variations in lipid profile parameters Pre and post values depicted as mean ± SD, p < 0.05 in bold. Due to a smaller sample size per group of ≤6, the non-parametric Wilcoxon signed-rank test was used for all comparisons. Pre-dosing represents the values of biochemical parameters at the end of the sixth week (post-induction). Post-dosing represents the values of biochemical parameters at the end of the 10th week (post-intervention). TC: total cholesterol; TG: triglyceride

Group	N	TC (mg/dL)			TG (mg/dL)		
		Pre	Post	p	Pre	Post	p
Negative control	6	199.3 ± 93.6	154.2 ± 91.4	0.5	131.3 ± 11.3	139.2 ± 14.4	0.03
Liv52	1	206	25	-	121	128	-
Glimepiride	5	83 ± 112.5	170 ± 107.4	0.4	118.6 ± 3.9	133.8 ± 16.2	0.1
Vildagliptin	6	163.8 ± 74.7	153.8 ± 177.5	0.9	120 ± 5	128 ± 17.7	0.4
Dapagliflozin	5	206.4 ± 16.4	103 ± 87	0.06	135.6 ± 9.1	125.2 ± 7.1	0.2
Pioglitazone	5	217.6 ± 137.4	113.4 ± 119.7	0.3	119.8 ± 2.9	131.8 ± 19.5	0.2

**Table 8:** Intra-group pre/post dosing variations in liver function parameters Pre and post values depicted as means ± SD; p < 0.05 in bold. Due to a smaller sample size per group of ≤6, the non-parametric Wilcoxon signed-rank test was used for all comparisons. Pre-dosing represents the values of biochemical parameters at the end of the sixth week (post-induction). Post-dosing represents the values of biochemical parameters at the end of the 10th week (post-intervention). AST: aspartate aminotransferase; ALT: alanine aminotransferase

Group	N	AST (U/L)			ALT (U/L)		
		Pre	Post	p	Pre	Post	P
Negative control	6	23 ± 24.2	11 ± 20.8	0.5	4.8 ± 6.4	4.5 ± 4.1	0.9
Liv52	1	42	44	-	0	0	-
Glimepiride	5	50.2 ± 4	10.7 ± 12.5	0.001	1 ± 2.2	0.4 ± 0.9	0.6
Vildagliptin	6	9.5 ± 7.3	1.2 ± 2.8	0.03	3.5 ± 5.2	0 ± 0	0.1
Dapagliflozin	5	25.4 ± 26.6	5 ± 11.2	0.2	1.6 ± 3	2.4 ± 3	0.4
Pioglitazone	5	40.8 ± 6.3	21.8 ± 19.6	0.2	0.6 ± 0.9	1.4 ± 2.2	0.6

**Figure 1:**
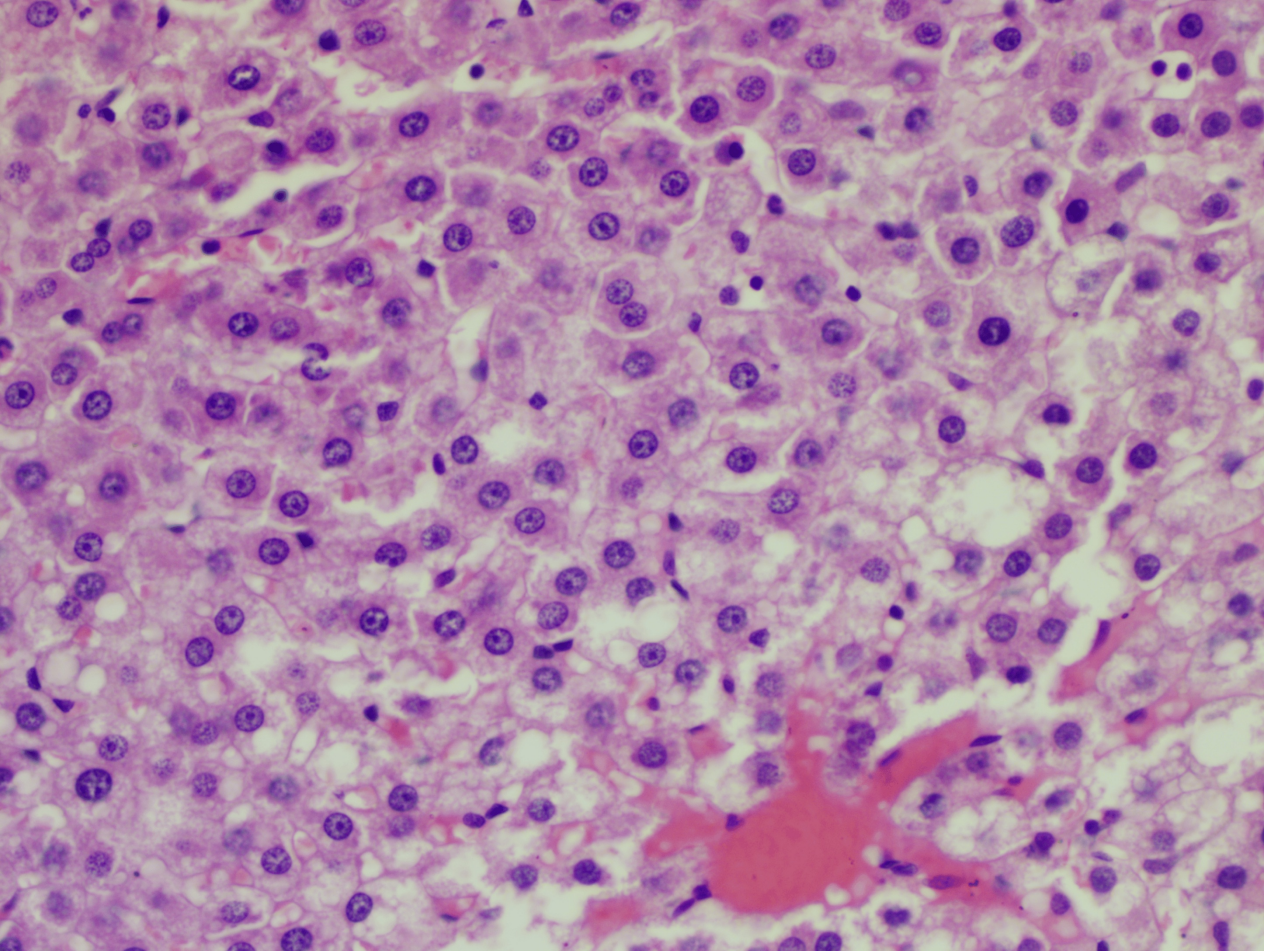
Slide of the negative control group Hematoxylin-eosin staining: 40x; lobular inflammation: +1; steatosis: +3; hepatocyte ballooning: +2; NAS: 6 NAS: non-alcoholic fatty liver disease (NAFLD) activity score

**Figure 2:**
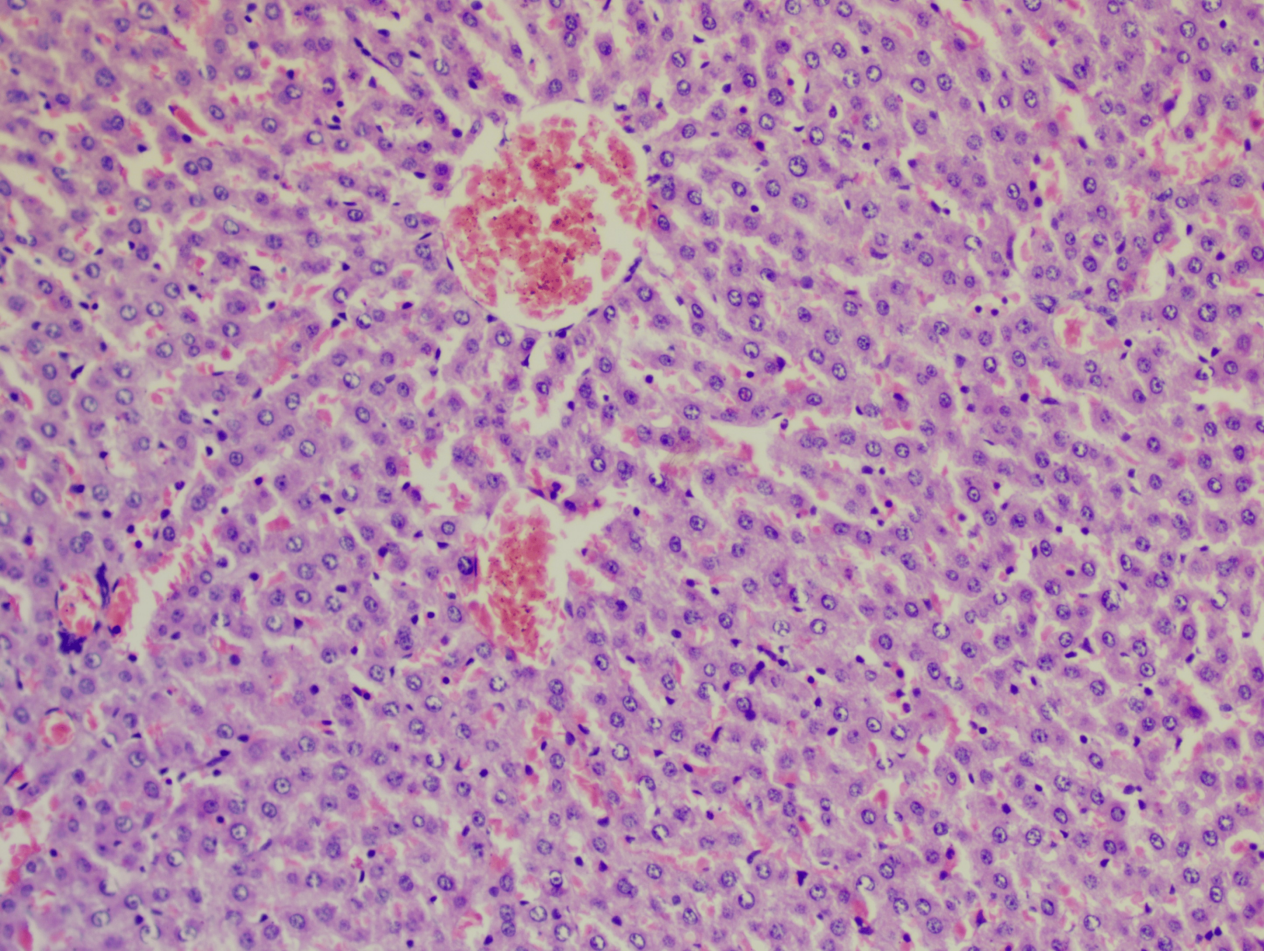
Slide of the glimepiride group Hematoxylin-eosin staining: 10x; lobular inflammation: +1; steatosis: +1; hepatocyte ballooning: 0; NAS: 2 NAS: non-alcoholic fatty liver disease (NAFLD) activity score

**Figure 3:**
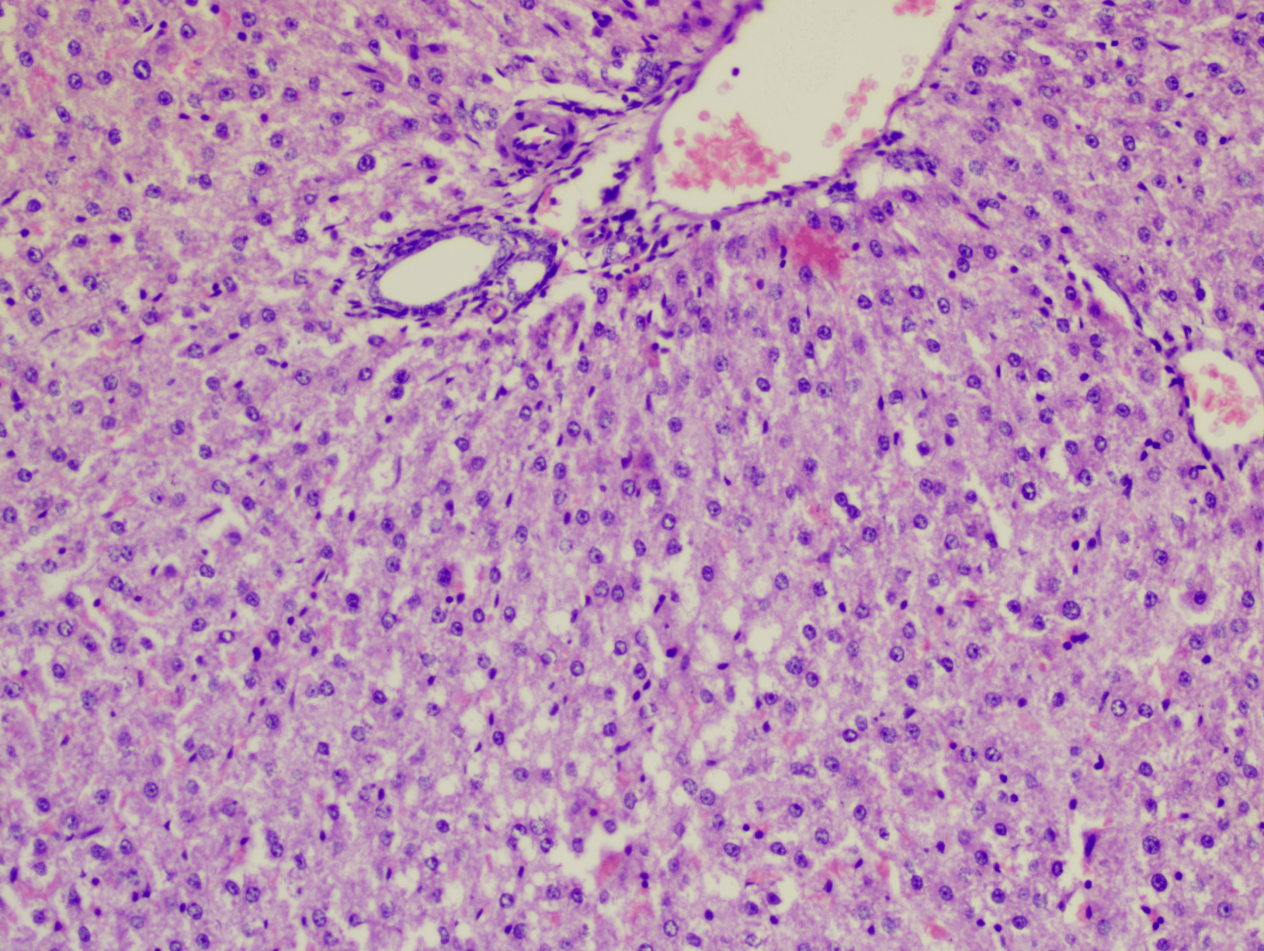
Slide of the dapagliflozin group Hematoxylin-eosin staining: 10x; lobular inflammation: +1; steatosis: +3; hepatocyte ballooning: +2; NAS: 6 NAS: non-alcoholic fatty liver disease (NAFLD) activity score

**Figure 4:**
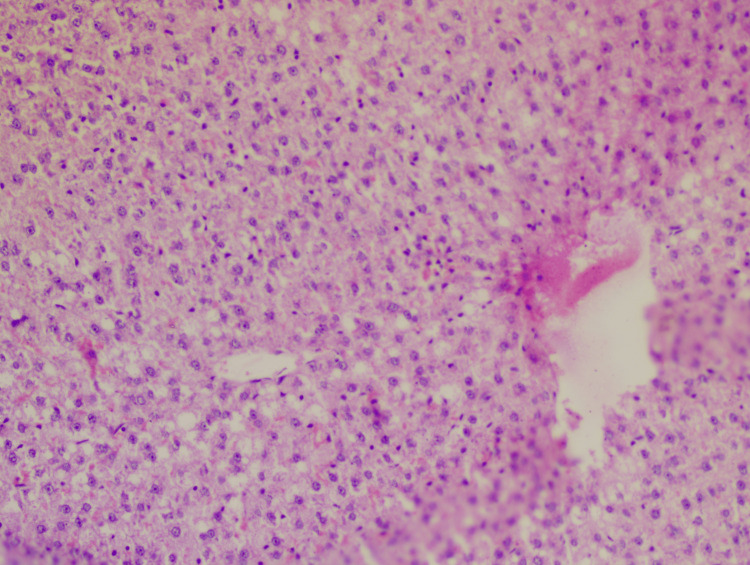
Slide of the vildagliptin group Hematoxylin-eosin staining: 10x; lobular inflammation: +1; steatosis: +2; hepatocyte ballooning: +2; NAS: 5 NAS: non-alcoholic fatty liver disease (NAFLD) activity score

**Figure 5:**
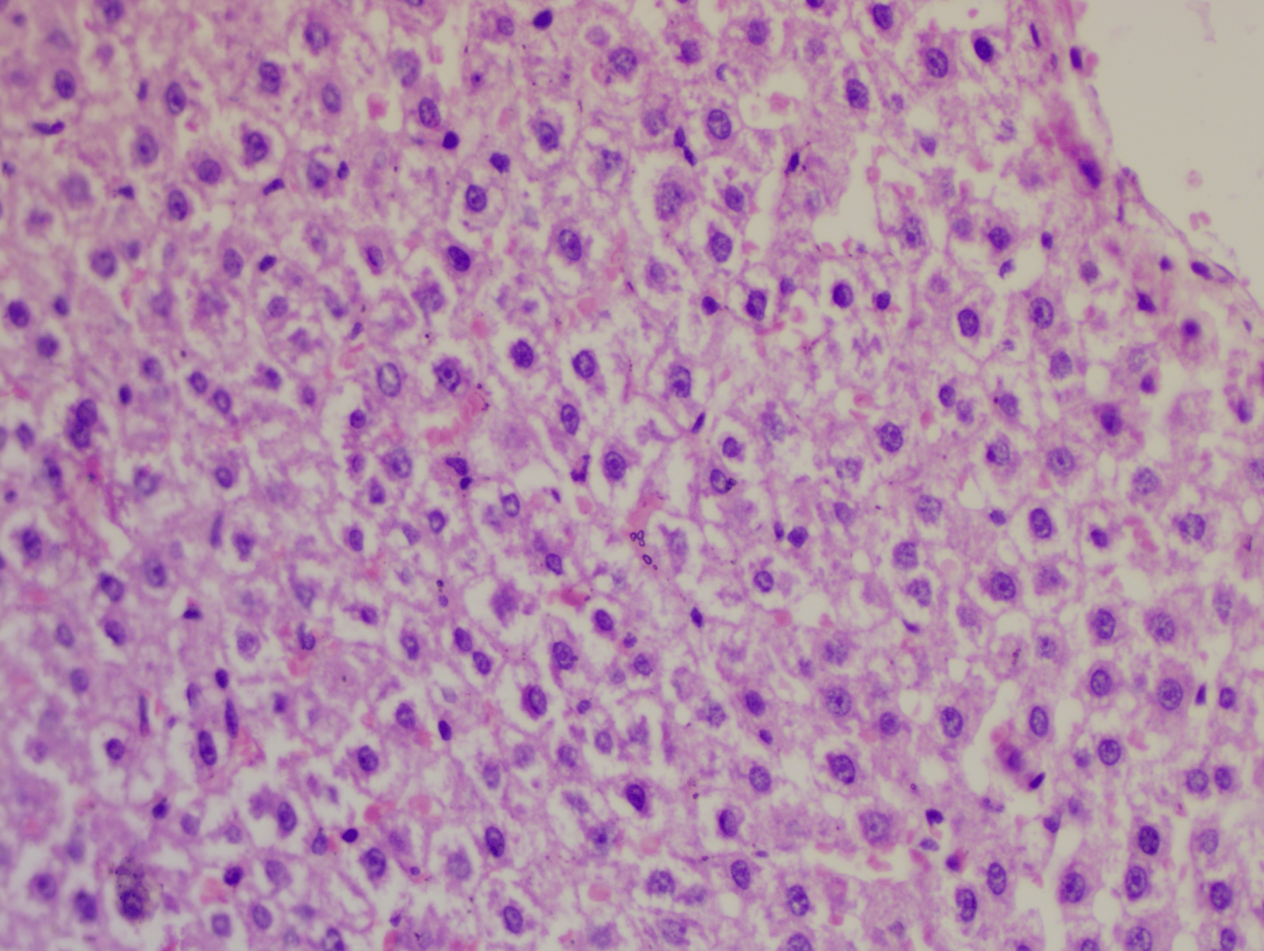
Slide of the Liv52 group Hematoxylin-eosin staining: 40x; lobular inflammation: +1; steatosis: +2; hepatocyte ballooning: +1; NAS: 4 NAS: non-alcoholic fatty liver disease (NAFLD) activity score

**Figure 6:**
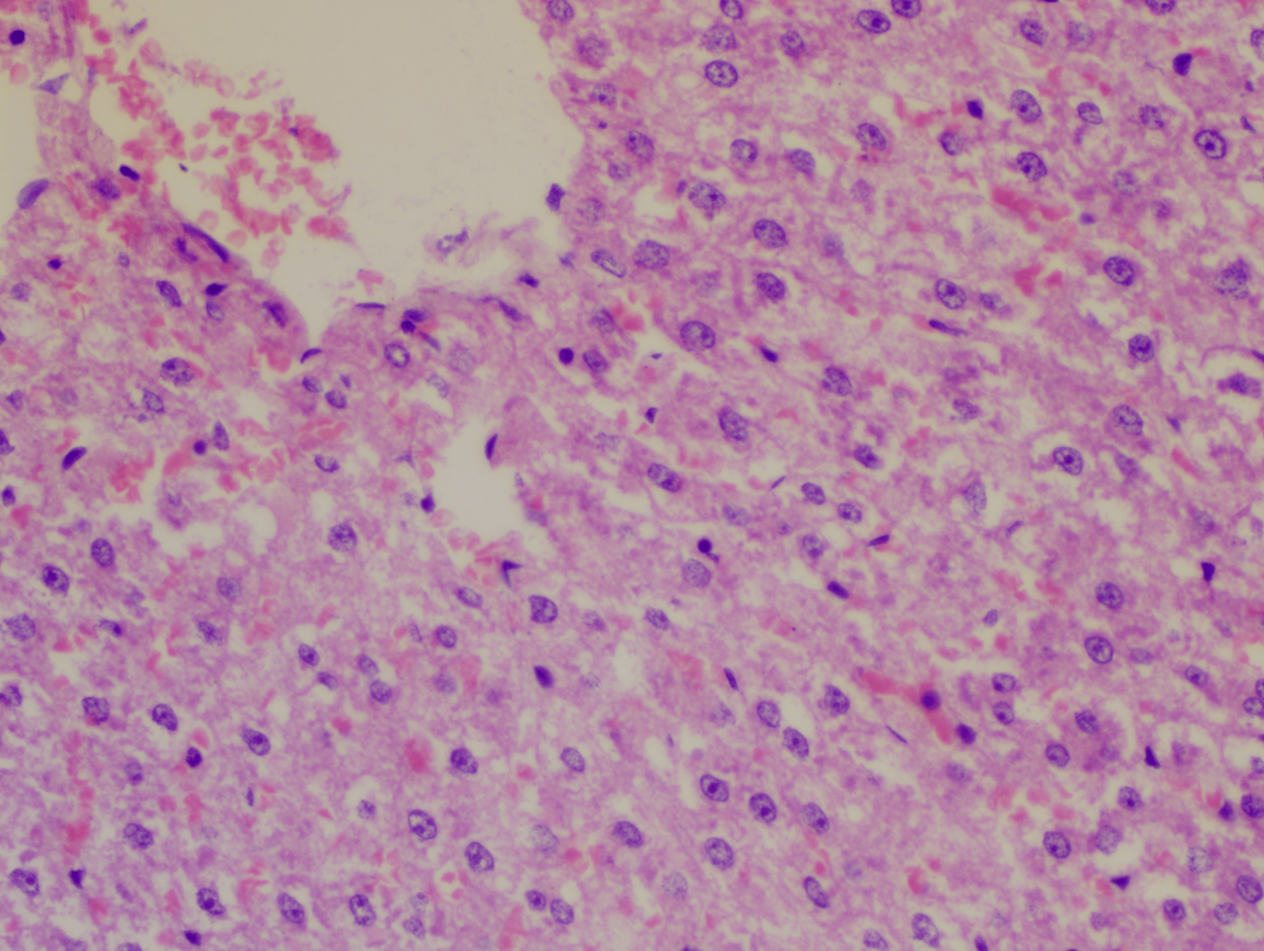
Slide of the positive control (pioglitazone) group Hematoxylin-eosin staining: 40x; lobular inflammation: 0; steatosis: +1; hepatocyte ballooning: +1; NAS: 2 NAS: non-alcoholic fatty liver disease (NAFLD) activity score

These results point to a “statistically significant amelioration of MASLD by virtue of AST reduction shown by glimepiride and vildagliptin, respectively” (p = 0.001 and 0.03). Finally, we did a histopathological analysis and NAS of isolated liver specimens from the rats. The results of that analysis along with representative images are as follows (Figures 1-6 and Table 9).

**Table 9:** NAS of different groups at the end of the 10th week (post-intervention) NC: negative control; G: glimepiride; L: Liv52; V: vildagliptin; D: dapagliflozin; PC: positive control (pioglitazone); NAS: non-alcoholic fatty liver disease (NAFLD) activity score (ranging from 0 to 8)

	NC	G	L	V	D	PC
NAS	6	2	4	5	6	2

Histopathological analysis of the isolated liver specimen slides of each individual group made it apparent that in comparison to the “NC,” which had the highest degree of MASLD (NAS: 6), the PC (pioglitazone) and glimepiride intervention groups exhibited the maximum reductions (to NAS of 2, respectively). The vildagliptin group, although portraying a statistically significant reduction in AST levels biochemically, caused only a modest reduction in the histopathological profile of MASLD (NAS of 5, just a one-point reduction compared to the NC, respectively). Hence, our study found that “glimepiride intervention led to statistically significant biochemical parameter (AST) reduction as well as a four-point reduction in NAS, similar to that shown by the PC (pioglitazone),” so it can be a potential candidate for MASLD amelioration in humans after thorough research in human clinical trials.

## Discussion

Our study was a novel pre-clinical study that sought to determine the effects of oral allopathic anti-hyperglycemic agents (pioglitazone, glimepiride, vildagliptin, and dapagliflozin) and an ayurvedic proprietary hepatoprotective agent (Liv52) in a head-to-head comparison amid Sprague-Dawley rats induced with MASLD using HFD, respectively. At the outset, the rat MASLD-HFD model we used (Sprague-Dawley, weighing 150-200 g, both male and female, aged 18-20 weeks, fed with HFD for six weeks) was in consonance with a variety of rat MASLD models used by other researchers worldwide.

The Sprague-Dawley rat HFD-MASLD model outshone the other strains in terms of fibrosis, hepatocellular damage, and central-vein blood-flow velocity reduction as noted by Rosenstengel et al. [15]. Results of their study were further corroborated by another study done by Stöppeler et al. whereby Lewis and Sprague-Dawley rats of both sexes were put on HFD for a similar three-week duration. Lewis rats subsequently developed microvesicular steatosis whereas Sprague-Dawley rats developed macrovesicular steatosis compounded with fibrosis [16].

We measured the baseline serum TC, TG, AST, and ALT levels in all our experimental rats to exclude any outliers. Thereafter, we gave HFD for a period of six weeks along with water ad libitum. Two rats (one of the Liv52 group and one of the PC - pioglitazone group) died during this six-week duration. It was followed by a repeat serum TC, TG, AST, and ALT ELISA estimation to confirm model induction biochemically.

On comparing the post-HFD sampling biochemical parameters’ values to those of the baseline after normality testing with the Shapiro-Wilk test followed by the paired t-test for parametric data and the Wilcoxon signed-rank test for non-parametric data, we found out that “HFD administration led to deranged TC, AST, and ALT values in the Sprague-Dawley rats,” thereby confirming model induction. Farooq et al. in their Sprague-Dawley rat liver cirrhosis MASLD model (using 2.5% urethane + dimethyl-sulfoxide for two weeks followed by carbon-tetrachloride mixed with peanut oil (50% v/v) for the next four weeks) also reported similar AST and ALT disruptions [17].

Looking at the MASLD histopathological profile in the NC group of our study (which had received only HFD for 10 weeks: six weeks model induction and four weeks experimental dosing phase), the NAS of 6 corroborates to “marked activity.” Similar findings of hepatocellular damage after HFD administration were also observed by Rosenstengel et al., Stöppeler et al., and Su et al., respectively, whereby HFD-induced fibrosis, central-vein blood-flow velocity reduction, macrovesicular steatosis, and NAS of 5 were reported after an HFD administration period of 10-12 weeks with no significant additional derangements seen if the HFD administration was continued till 24 weeks, respectively [15,16,18].

Coming to the results shown by individual intervention groups after the administration of the drug by oral gavage along with HFD for four weeks, the glimepiride group (G) exhibited mortality of one out of the six rats during the dosing period, and the comparisons of remnant rats’ (n = 5) biochemical parameters vis-à-vis pre and post dosing show increased serum TC values (83 mg/dL vs. 170 mg/dL, p = 0.4) and serum TG values (118.6 mg/dL vs. 133.8 mg/dL, p = 0.1), which although statistically insignificant are novel findings for TC and can be attributed to the weight gain seen in these rats. Glimepiride intervention leading to increased serum TG has also been reported by Kinoshita et al. (154.1 ± 17.0 mg/dL vs. 156.8 ± 16.2 mg/dL) [19].

Also, we observed reduced serum AST values (50.2 U/L vs. 10.7 U/L, p = 0.001) and serum ALT values (1 U/L vs. 0.4 U/L, p = 0.6), respectively. The statistically significant AST reduction along with marginal ALT reduction points to an amelioration of hepatic transaminase enzymes, an important predictor of MASLD activity.

Additionally, the glimepiride group exhibited a four-point reduction in NAS compared to the NC (NAS: 2 vs. 6), which was comparable to the reduction shown by the PC - pioglitazone. Similar findings were also reported by Tian et al.: reduction in AST and ALT (39.26 ± 12.06 IU/L vs. 32.62 ± 8.63 IU/L and 46.79 ± 11.85 vs. 33.51 ± 13.68 IU/L) [20], Takeshita et al.: reduction in AST and ALT (30 IU/L vs. 25 IU/L and 48 IU/L vs. 38 IU/L) along with amelioration of NAS (30% improvement in steatosis, p = 0.06; 25% improvement in hepatocellular ballooning, p = 0.02; and 15% improvement in lobular inflammation, p = 0.7, respectively) [8], Kinoshita et al.: reduction in ALT (45.3 ± 4.6 IU/L vs. 44.3 ± 4.7 IU/L) [19], and Li et al.: reduction in AST and quantitative detection of liver steatosis (28.88 ± 14.08 U/L vs. 22.03 ± 10.27 U/L and 21.11 ± 8.85% vs. 20.13 ± 8.18%), respectively [21]. Hence, the data obtained from our study match clinical human data revealed from other studies in glimepiride causing a reduction in AST, ALT, and NAS along with increased TC and TG levels, respectively.

Moving on to the next allopathic drug intervention group of our study, vildagliptin (V) exhibited no intra-group rat mortality, and all six rats reached the end stage of our study. Comparisons of biochemical parameters vis-à-vis pre and post dosing show increased serum TG levels (120 mg/dL vs. 128 mg/dL, p = 0.4), which although statistically insignificant is a novel finding of our study and could be attributed to the differences in metabolic pathways between humans and rodents as the amelioration of serum TG levels by vildagliptin has been observed in human clinical trials. Furthermore, we observed a reduction in serum TC (163.8 mg/dL vs. 153.8 mg/dL, p = 0.9), AST (9.5 IU/L vs. 1.2 IU/L, p = 0.03), and ALT (3.5 IU/L vs. 0 IU/L, p = 0.1) values along with a marginal one-point reduction in NAS by vildagliptin in comparison to the NC (NAS: 5 vs. 6).

Similar findings were also reported by Mookkan et al.: reduction in TC, AST, and ALT (128.5 ± 13.36 mg/dL vs. 99.57 ± 18.53 mg/dL, 119.5 ± 13.74 IU/L vs. 92.72 ± 8.96 IU/L, and 58.67 ± 7.04 IU/L vs. 47.33 ± 8.7 IU/L) along with insignificant improvement of NAS [22], Hussain et al.: reduction in TC, AST, and ALT (252.6 ± 24.4 mg/dL vs. 220.6 ± 20.2 mg/dL, p = 0.03; 63.5 ± 10.5 IU/L vs. 41.5 ± 9.6 IU/L, p = 0.002; and 78.2 ± 17.2 IU/L vs. 48.6 ± 14.8 IU/L, p = 0.04) along with regression in fatty liver grading (grade 1: 100% vs. 37.5%, grade 2: 58.8% vs. 12.5%, and grade 3: 40% vs. 20%, respectively) [23], and ElKabbany et al.: reduction in TC and hepatic steatosis index (p < 0.001 for both) [24]. Hence, the data obtained from our study match clinical human data as well as mouse-based pre-clinical data revealed from other studies in vildagliptin causing a reduction in TC, AST, ALT, and NAS along with increased TG levels, respectively.

The last allopathic drug intervention group was of dapagliflozin (D). It exhibited mortality of one out of the six rats during the dosing period, and the comparisons of remnant rats’ (n = 5) biochemical parameters vis-à-vis pre and post dosing show increased serum ALT levels (1.6 IU/L vs. 2.4 IU/L, p = 0.4), which although statistically insignificant is a novel finding of our study and could be attributed to the differences in metabolic pathways between humans and rodents as the amelioration of serum ALT levels by dapagliflozin has been observed in human clinical trials.

Also, we observed a reduction in serum TC, TG, and AST values (206.4 mg/dL vs. 103 mg/dL, p = 0.06; 135.6 mg/dL vs. 125.2 mg/dL, p = 0.2; and 25.4 IU/L vs. 5 IU/L, p = 0.2, respectively). Although statistically insignificant, the results point to an amelioration of lipid parameters along with hepatic transaminase enzymes, displaying an amelioration of MASLD biochemically. Interestingly, the dapagliflozin group exhibited no reduction in NAS histopathologically post-intervention in comparison to the NC (NAS 6 vs. 6).

Similar findings were also reported by Gao et al.: reduction in serum TC, TG, and AST levels (4.5 ± 0.2 mmol/L vs. 3.9 ± 0.1 mmol/L, p < 0.001; 1.75 mmol/L vs. 1.34 mmol/L, p < 0.001; and 24.0 ± 2.1 IU/L vs. 19.0 ± 0.9, p = 0.007) [25]; Said Ahmed et al.: reduction in serum TC, TG, and AST levels (112 ± 4.7 mg/dL vs. 45 ± 2.7 mg/dL, p = 0.001; 130 ± 3.45 mg/dL vs. 48.5 ± 6.1 mg/dL, p = 0.001; and 102.9 ± 12.7 IU/L vs. 33.2 ± 7.4 IU/L, p = 0.001) in albino rats [26]; Zhao et al.: reduction in serum TC and TG (p < 0.05 for both) in db/db mice [27]; Cho et al.: reduction in serum TC level (p = 0.04) [28]; Li et al.: reduction in serum TG level (p < 0.05) in ZDF rats [29]; Das et al.: reduction in serum TG and AST levels (172 ± 21.41 mg/dL vs. 147.5 ± 22 mg/dL, p = 0.0001 and 55.88 ± 20.92 IU/L vs. 49.52 ± 16.61 IU/L, p = 0.0001) [30]; Tobita et al.: reduction in serum AST levels (42.3 ± 1.6 IU/L vs. 37.3 ± 13.8 IU/L, p < 0.05) [31]; Kinoshita et al.: reduction in serum TG and AST levels (173.2 ± 26.0 mg/dL vs. 140.9 ± 10.4 mg/dL and 38.8 ± 4.1 IU/L vs. 30.2 ± 3.1 IU/L) [19]; Gastaldelli et al.: reduction in serum AST levels (22.7 ± 11.4 IU/L vs. 20.25 ± 4.3 IU/L, p = 0.0005) [32]; Arase et al.: reduction in serum TG and AST values (median (range): 154 (70-300) mg/dL vs. 119 (57-224) mg/dL, p = 0.04 and 30 (15-83) IU/L vs. 21 (10-63) IU/L, p = 0.007) [33]; and Eriksson et al.: reduction in serum AST levels (p < 0.05) [34], respectively. Hence, data obtained from our study match clinical human data as well as mouse and rat-based pre-clinical data revealed from other studies in dapagliflozin causing a reduction in TC, TG, and AST along with increased ALT levels, respectively.

Moving on to the ayurvedic hepatoprotective agent Liv52 (L) group, we saw quite distinct outcomes in our pre-clinical study. Out of the six rats assigned to this group at the outset, one rat died while in the model induction phase itself (HFD feeding), while four rats died within a week of drug administration. Liver histopathology of the deceased rats revealed features like congested central vein, dilated and congested sinusoids, microvesicular steatosis, peri-portal inflammation, hepatocyte ballooning, and focal centri-lobular necrosis. Interestingly, available clinical and pre-clinical data about the safety of Liv52 point to no significant hepatic damage caused by this drug (Ali et al., Yildirim et al., Cimen et al., Al-Awthan and Salem Bahattab, Medhekar et al., Maity and Mandal, and Ghosh et al.) [35-41]. Hence, further clinical studies need to be carried out to shed more light on this area.

For the one rat that did indeed survive the month-long drug intervention, biochemical parameters vis-à-vis pre and post dosing show increased serum TG and AST levels (121 mg/dL vs. 128 mg/dL and 42 IU/L vs. 44 IU/L), which points to a finding corroborative with liver damage. Also, we observed no change in serum ALT values (0 IU/L vs. 0 IU/L) and a reduction in serum TC levels (206 mg/dL vs. 25 mg/dL). Finally, the Liv52 group (n = 1) exhibited a two-point reduction in NAS histopathologically post-intervention in comparison to the NC (NAS 4 vs. 6).

Similar findings were also reported by Yildirim et al.: improved hepatic histopathological profile with Liv52 pre-treatment in doxorubicin-induced hepatotoxicity in rats [36]; Cimen et al.: Liv52 ameliorated liver ischemia-reperfusion damage histopathologically in male albino Wistar rats [37]; Al-Awthan and Salem Bahattab: Liv52 ameliorated histopathological features of vasodilation, hemorrhage, cytoplasmic vacuolization, inflammation, and nuclear pyknosis in dimethoate-induced hepatotoxicity in male guinea pigs [38]; Maity and Mandal: amelioration of MASLD based on ultrasonographic findings (hepatomegaly and infiltration: 3.53 ± 0.84 units vs. 2.21 ± 0.80 units, p < 0.0003) [40]; and Ghosh et al.: reduction in serum TC levels (204.30 ± 18.23 mg/dL vs. 181.30 ± 15.26 mg/dL, p < 0.0001) and amelioration of MASLD based on ultrasonographic findings (1.58 ± 0.64 units vs. 0.66 ± 0.56 units, p < 0.0001) [41], respectively. Hence, data obtained from our study match clinical human data as well as rat and guinea pig-based pre-clinical data revealed from other studies in Liv52 causing a reduction in TC and NAS along with increased TG and AST levels as well as no significant changes in ALT levels, respectively. The high intra-group mortality seen with Liv52 needs further investigation in human clinical studies.

Coming to the last group of our study, the PC (pioglitazone) group, it exhibited mortality of one out of the six rats during the HFD-feeding induction phase, and the comparisons of remnant rats’ (n = 5) biochemical parameters vis-à-vis pre and post dosing show increased TG and ALT (119.8 mg/dL vs. 131.8 mg/dL, p = 0.2 and 0.6 IU/L vs. 1.4 IU/L, p = 0.6), which although statistically insignificant is a novel finding of our study and could be attributed to the differences in metabolic pathways between humans and rodents as the amelioration of serum TG and ALT levels by pioglitazone has been observed in human clinical trials.

Also, we observed a reduction in serum TC and AST values (217.6 mg/dL vs. 113.4 mg/dL, p = 0.3 and 40.8 IU/L vs. 21.8 IU/L, p = 0.2), respectively. Although statistically insignificant, the results point to an amelioration of lipid parameters along with hepatic transaminase enzymes, displaying an amelioration of MASLD biochemically. Furthermore, the pioglitazone group exhibited the highest (four-point) reduction in NAS in comparison to the NC (NAS 2 vs. 6), which is consistent with its usage as a PC in our study.

Similar findings were also reported by Cusi et al.: improvement in NAS, including fibrosis score (NAS change = -0.5, p = 0.04) [42]; Kinoshita et al.: reduction in serum AST levels (34.1 ± 3.9 IU/L vs. 26.9 ± 2.0 IU/L) [19]; Aithal et al.: reduction in NAS (hepatic steatosis component, p = 0.001; hepatocellular injury component, p = 0.005; lobular inflammation component, p = 0.04; Mallory-Denk bodies component, p = 0.004; and fibrosis component, p = 0.006) [43]; Sanyal et al.: reduction in serum AST levels and NAS (change = -20.4 IU/L, p < 0.001; steatosis improvement in 69% subjects, p < 0.001; lobular inflammation improvement in 60% subjects, p = 0.004; and total NAS mean change: -1.9, p < 0.001, respectively) [44]; and Belfort et al.: reduction in serum AST levels and NAS (47 ± 15 IU/L vs. 28 ± 7 IU/L, p < 0.001; steatosis improvement in 65% subjects, p < 0.001; ballooning necrosis improvement in 54% subjects, p < 0.001; lobular inflammation improvement in 65% subjects, p < 0.001; improvement in combined necro-inflammation scores: 85%, p < 0.001; and fibrosis improvement: 46%, p = 0.002) [45], respectively. Hence, data obtained from our study match clinical human data revealed from other studies in pioglitazone causing a reduction in TC, AST, and NAS along with increased TG and ALT levels, respectively. These results justify the usage of pioglitazone as a PC in our study for the amelioration of MASLD.

Study limitations

As our study was a pre-clinical study, it could generate only preliminary evidence of the efficacy of the aforementioned allopathic and ayurvedic medicines for the treatment of MASLD. Further human clinical trials focusing on the efficacy and safety of these drugs in MASLD with or without type 2 diabetes mellitus (T2DM) need to be conducted to obtain definitive evidence for the suitability of usage of these drugs in humans. The human clinical trials pertaining to this should include a wider array of diagnostic modalities, ranging from a complete liver function test profile, lipid profile, and hemogram to liver fibroscan for stiffness and controlled attenuation parameter (CAP) values, respectively. Also, we carried out the study using only one dose of each drug being given daily for four weeks; further studies can assess the safety and efficacy of a multitude of doses of each drug, respectively.

## Conclusions

After analyzing the biochemical and histopathological changes brought about by the drug interventions on the HFD-MASLD Sprague-Dawley rat model, we conclude that glimepiride, which exhibited AST and ALT reductions along with a four-point NAS reduction, offers itself as a promising drug candidate for human clinical trials for MASLD. Vildagliptin, on the other hand, exhibited TC, AST, and ALT reductions but a less promising one-point NAS reduction, respectively.
